# Histoplasmosis Presenting as Granulomatous Hepatitis: Case Report and Review of the Literature

**DOI:** 10.1155/2014/879535

**Published:** 2014-06-09

**Authors:** Nancy A. Rihana, Manasa Kandula, Ana Velez, Kumud Dahal, Edward B. O'Neill

**Affiliations:** ^1^Department of Infectious Diseases, University of South Florida, 1 Tampa General Circle G323, Tampa, FL 33606, USA; ^2^University of South Florida, Tampa, FL 33606, USA; ^3^Department of Infectious Diseases, University of South Florida, Tampa, FL 33606, USA; ^4^Department of Infectious Diseases, University of Illinois, One Illini Drive, Peoria, IL 61605-2576, USA; ^5^Department of Pathology, Tampa General Hospital, Tampa, FL 33606, USA

## Abstract

*Background*. *Histoplasma capsulatum* is the most common endemic mycosis in the United States and is a frequent cause of opportunistic infection in immunodeficient hosts. Histoplasmosis is most often self-limiting and goes unrecognized in the immunocompetent population but can progress to disseminated histoplasmosis in patients with an impaired immune system. Liver involvement as a part of disseminated histoplasmosis which usually originates in the lung is well known. However, extrapulmonary hepatic histoplasmosis as a primary manifestation is extremely rare. *Case Presentation*. We report a rare case of histoplasmosis that presented as persistent fever and abnormal liver function tests in a 66-year-old female with rheumatoid arthritis, receiving infliximab. *Conclusion*. Emphasizing histoplasmosis as a major cause of acute granulomatous hepatitis and fever of unknown origin in cell mediated immunodeficient population, this case highlights the need for high index of suspicion and the importance of prompt diagnosis since any delay of treatment can be life threatening in this population.

## 1. Background

*Histoplasma capsulatum var. capsulatum* is the most common cause of endemic mycosis in the US. It remains a frequent cause of opportunistic infection in patients with compromised immune status, iatrogenic or related to human immunodeficiency virus (HIV). The vast majority of histoplasmosis is subclinical. It either goes unrecognized or presents as a mild influenza like illness, and the small proportion who develop progressive disseminated histoplasmosis are the immunosuppressed population [1]. Liver involvement as a part of disseminated histoplasmosis is well known. However, liver infection as primary manifestation of histoplasmosis, without evidence of primary lung involvement is rare. Here we report a case of histoplasmosis presenting with acute granulomatous hepatitis.

## 2. Case Presentation

The patient is a 66-year-old Caucasian female who presented to our hospital with three weeks history of fever of unknown origin with no specific pattern, associated with nausea, vomiting, fatigue, and pruritus. The patient was already admitted twice to an outside hospital within the last month and underwent extensive diagnostic workup. She had a bronchoscopy with BAL studies as well as lung biopsy of an incidental 1.6 cm right lower lobe lung nodule, seen on imaging studies, with no definite diagnosis.

Her past medical history is significant for 16 years history of rheumatoid arthritis, for which she has been on methotrexate for the last 8 years and infliximab for the last 6 years. At the outside hospital, she was found to have elevated liver enzymes with subsequent discontinuation of methotrexate and infliximab. The patient was started on prednisone at a dose of 40 mg daily.

Upon arrival to our hospital, the patient was found to have a fever of 103°F with chills and tachycardia, along with painless jaundice, fatigue, nausea, and vomiting.

She denies any history of sick contacts, unpasteurized milk, or undercooked meat intake. Upon further questioning, she reports that her fever started only two days after a long road trip with her husband, from Michigan to Florida. On their way, and 11 days prior to her symptoms started, they stopped at Pittsburg, Kansas, where they stayed for 6 days. The patient endorsed horseback riding in Pittsburg but, however, denied any sick contacts.

Her examination was notable for an awake, alert, and oriented patient with icteric sclerae. Her respiratory rate was 18 breaths per minute and was saturating 98% on room air. There was no hepatosplenomegaly or abdominal tenderness and the rest of the examination was normal.

Laboratory examination revealed hemoglobin of 12.3 g/dL, white cell count of 7.9 with 40% granulocytes, 43% lymphocytes and 7% monocytes, and platelets of 230. Liver function tests revealed alanine aminotransferase 252 IU/L (0–60 IU/L), aspartate aminotransferase 173 IU/L (0–46 IU/L), alkaline phosphatase of 375 IU/L (44–147 IU/L), total bilirubin 4.2 mg/dL (0-1 mg/dL), corrected calcium 8.7 mg/dL (8.5–10.5 mg/dL), PT 11.4 (9–12.5 sec), total protein of 7 mg/dL, and an albumin of 2.5 mg/dL. Autoimmune markers, including serum antimitochondrial antibodies, were negative, except for RF and anti-CCP. Blood cultures and urine culture remained negative.

Ultrasound of the abdomen was normal. The chest X-ray again revealed a right lower lobe pulmonary nodule. Computed tomography (CT) of the chest showed a 1.3 cm right lower lobe pulmonary nodule which corresponds to the abnormality seen on chest X-ray. CT of the abdomen showed normal appearance and size of liver, pancreas, spleen, and adrenals.

Outside records were reviewed and bronchial washings had grown only* Candida albicans* on routine bacterial and fungal cultures. Cytology was consistent with mixed inflammatory cells with no evidence of microorganism on Grocott's methenamine silver (GMS) stain. Right lower lobe nodule CT guided needle biopsy was only consistent with focal acute and chronic inflammation, along with scattered yeast elements consistent with candida species, with no evidence of granulomas or tumor.

CMV PCR and hepatitis serologies were negative. EBV blood PCR came back positive with 1959 copies/cc (IgG VCA positive, IgM VCA negative). Serum RPR and QuantiFERON test for tuberculosis were negative. Serum cryptococcal antigen from outside hospital was negative.

**Figure 1:**
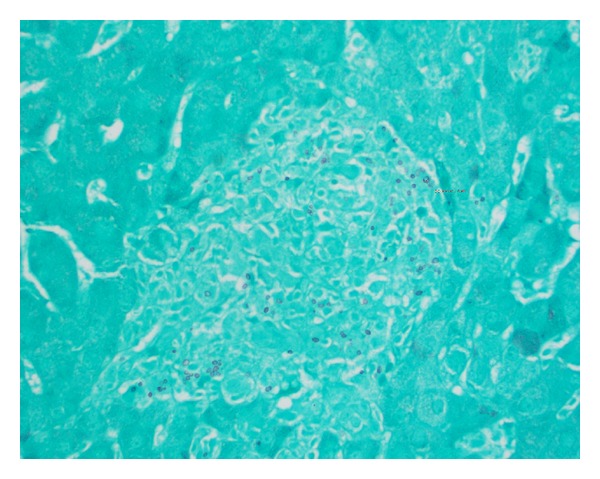
Liver tissue with Grocott's methenamine silver stains (GMS) showing round to ovoid 2–4 micrometers narrow based budding fungal organisms.

Empiric antibiotics consisting of vancomycin and piperacillin-tazobactam were discontinued after final blood cultures reported no growth and patient remained febrile. Transthoracic echocardiogram was compatible with grade 1 diastolic dysfunction, normal ejection fraction, and no vegetation. An ultrasound guided liver biopsy, done on hospital day two, was consistent with granulomatous hepatitis with negative acid fast stain, and subsequently steroids were restarted.* Cryptococcal* antigen and* Brucella*,* Bartonella* and Q fever serologies were all negative. Liver biopsy immunohistochemical stain with* in situ* hybridization studies was also negative for EBV. On hospital day five, after additional stains on liver specimen were performed, pathological examination reported that fungal organism was identified within areas of granulomatous inflammation, largely within histiocytes on hematoxylin and eosin staining. They were further described as round to ovoid 2–4 micrometers narrow based budding fungal organisms on GMS staining suggestive of histoplasmosis (Figure 1). Three days later, serum histoplasma antigen, by quantitative MVista Histoplasma antigen enzyme immunoassay (EIA) detection test, reported strongly positive with titers above the limit of quantification, above 19 ng/mL.

Meanwhile, patient clinical condition deteriorated and progressed into a respiratory failure with bilateral interstitial infiltrates on chest imaging requiring mechanical ventilation. Intravenous liposomal amphotericin B at a dose of 3 mg/kg q 24 hrs was ultimately started. Fungal culture from bronchoalveolar lavage eventually grew* Histoplasma capsulatum*. The patient became afebrile on day five of amphotericin and her jaundice started to resolve. Her chest X-ray cleared and was extubated. The patient was switched on day seven to itraconazole secondary to acute kidney injury. Antifungal therapy was subsequently switched to voriconazole once she developed a bleeding peptic ulcer disease requiring intravenous proton pomp inhibitor while being on itraconazole.

Patient's liver function tests completely normalized in six weeks.

## 3. Discussion

Granulomatous hepatitis is an important cause of fever of unknown origin in up to 13% of patients [2]. Granulomatous hepatitis may be the first clue to an ongoing systemic disease. This entity has a broad differential that goes from autoimmune disorders like sarcoidosis and primary biliary cirrhosis to malignancy, drugs, and systemic infections like tuberculosis, fungal infections, Q fever, and brucellosis or it may be idiopathic [3]. Clinically these patients are usually symptomatic with elevated liver function tests and fever of unknown origin [4].

Histoplasmosis is a thermal dimorphic fungus found most commonly in soil along the Ohio and Mississippi River Valleys, in the central, mid-Atlantic, and south-central states, and from Alabama to southwest Texas. When soil is disturbed by excavation or construction, the spores become airborne and are inhaled [1]. In highly prevalent areas, such as Indianapolis and Kansas City, more than 80% of the population has been exposed to histoplasma through inhalation of airborne spores. Once in the alveoli they convert to a yeast form, which is the tissue invasive form. The current multiplying yeasts are phagocytosed by alveolar macrophages that are initially incapable of killing the fungus. The ingested yeasts multiply inside the macrophages and are spread throughout the body via the lymphatics during the preimmune phase of the illness to organs rich in reticuloendothelial cells [5]. Hematogenous dissemination usually occurs during the first 2 weeks before cellular immunity develops [6]. In an immunocompetent host, once adequate cell-mediated immunity develops, the infection is eliminated by cytokines including interleukin-12 and interferon gamma that help “armed” macrophages in either killing the organism or halt its progression by forming a calcified granuloma [7]. In immunosuppressed individuals, especially with defective cell mediated immunity, like our patient, these defense mechanisms are impaired leading to reactivation of old foci or a progressive dissemination involving the extrapulmonary tissues [8]. The typical incubation time is 7–21 days [1].

A variety of well-known conditions can predispose to disseminated histoplasmosis, that is, AIDS, immunosuppressive medications such as glucocorticoids, antirejection therapies in solid organ transplantation or TNF alpha inhibitors therapies, primary immunodeficiency, and extremes of age [9–11].* Histoplasma capsulatum* is a well-recognized but uncommon cause of granulomatous hepatitis. Liver often is involved in the course of progressive disseminated disease usually originating in the lungs. Patients with primarily liver involvement present with nonspecific symptoms like fever of unknown origin, nausea, vomiting, fatigue, weight loss, and elevation of liver function tests as seen in our patient and previously reported cases [12, 13].

A diagnosis of disseminated histoplasmosis can be made using various methods like antigen detection, cultures, serology, or direct microscopy. The gold standard is the recovery of the organism from biologic extrapulmonary material [5]. In our case, the granuloma on the histopathologic exam and the microscopic demonstration of histoplasma provided a rapid and definitive proof of dissemination. Fungal culture of the tissue samples can provide a definitive diagnosis as well, but it will take at least 1 month to ensure isolation, with a 80% sensitivity only if multiple specimens are submitted [14]. However, as a quick and reliable assay, tests for serum and urine histoplasma polysaccharide antigen remain the mainstay of diagnosis. Based on multicenter evaluation of MVista Histoplasma antigen EIA of 218 patients with histoplasmosis, antigenuria was detected in 92% of PDH (progressive disseminated histoplasmosis) cases, including 95% of those with AIDS, 93% of those with other immunocompromising conditions, and 73% of patients not known to be immunocompromised. Among patients with PDH for whom serum and urine samples were tested, antigenuria was detected in 97% and antigenemia in 100% [15]. Because 2 to 6 weeks may be required for the appearance of antibodies, serology is less useful for patients who have severe acute infection and in immunocompromised patients, who mount a poor response [11]. Serology sensitivity is only 70% in the immunocompromised population and particularly only 20% for patients taking TNF alpha inhibitors [16]. Imaging studies lack sensitivity and specificity and have very limited role in the diagnosis of granulomatous hepatitis.

Prompt diagnosis and initiation of antifungal therapy are crucial in patients with impaired cell mediated immunity with fatality rate of 100% if left untreated [1]. The two agents most commonly used for the treatment of histoplasmosis include amphotericin B and itraconazole. For moderately severe to severe disease, liposomal amphotericin B (3 mg/kg daily) is recommended for 1-2 weeks, followed by oral itraconazole (200 mg 3 times daily for 3 days and then 200 mg twice daily for a total of at least 12 months) [17]. Itraconazole can be used initially in patients with mild-to-moderate disease. Lifelong suppressive therapy with itraconazole (200 mg daily) may be required in immunosuppressed patients if immunosuppression cannot be reversed [17]. Fluconazole is not as active as itraconazole against* Histoplasma capsulatum*. Voriconazole and Posaconazole have good in vitro activity against histoplasma but are not commonly used. Resistance can emerge during voriconazole therapy but has not been described with Posaconazole [18]. Antigen levels in urine can be used to monitor a patient response to treatment, even though most of these reported studies were done with AIDS patients [11]. Antigen levels decline by the end of 12 weeks; most of the decline was seen in the first 2 weeks of therapy, more rapidly in serum than in urine [19]. An increase in antigen concentration raises concern for relapse or treatment failure and further evaluation of the adequacy of treatment is indicated [11].

Our patient had 6-year history of cell mediated immunodeficiency as a well-known risk factor for disseminated histoplasmosis; this long interval makes this presentation more consistent with a recent exposure and spore inhalation while horseback riding in an endemic area rather than a reactivation of prior infection. The pathogenesis behind early liver involvement and lung sparing in the initial presentation remains unexplained. This finding has been well reported with tuberculosis after infliximab therapy, where the majority of patients had extrapulmonary tuberculosis (56%) [20]. Moreover, there is always the possibility of subclinical primary lung involvement that was underdiagnosed in this patient. In the latter case, the ability of the patient to mount a well formed granulomatous reaction selectively in the liver and failure of granuloma formation in the lung remains unexplained. In addition, her clinical deterioration after stopping TNF blockers raises the possibility of immune reconstitution inflammatory syndrome (IRIS) [16]. High clinical suspicion is critical for early diagnosis because if was left untreated; the infection can disseminate further over the next 2 to 12 weeks leading to a high fatality rate [21].

## 4. Conclusion

Disseminated histoplasmosis presenting as granulomatous liver disease is rare. High index of suspicion is warranted in patients with impaired cell mediated immunity, who present with fever of unknown origin and elevated liver function tests, especially if the patient has resided or visited an endemic area. Rapid diagnosis by antigen testing or by histopathologic examination is essential to facilitate institution of appropriate antifungal therapy and minimize mortality.
